# Examining provincial PrEP coverage and characterizing PrEP awareness and use among gay, bisexual and other men who have sex with men in Vancouver, Toronto and Montreal, 2017–2020

**DOI:** 10.1002/jia2.26017

**Published:** 2022-10-28

**Authors:** Jordan M. Sang, Kate McAllister, Lu Wang, Justin Barath, Allan Lal, Abbie Parlette, Syed W. Noor, Herak Apelian, Shayna Skakoon‐Sparling, Mark Hull, David M. Moore, Joseph Cox, Trevor A. Hart, Gilles Lambert, Daniel Grace, Jody Jollimore, Robert S. Hogg, Nathan J. Lachowsky

**Affiliations:** ^1^ British Columbia Centre for Excellence in HIV/AIDS Vancouver British Columbia Canada; ^2^ School of Public Health and Social Policy University of Victoria Victoria British Columbia Canada; ^3^ Ryerson University Toronto Ontario Canada; ^4^ School of Human Sciences Louisiana State University Shreveport Shreveport Louisiana USA; ^5^ McGill University Montréal Quebec Canada; ^6^ Direction régionale de santé publique ‐Montréal CIUSSS Centre‐Sud‐de‐l'Ile‐de‐Montréal Montréal Quebec Canada; ^7^ University of British Columbia Vancouver British Columbia Canada; ^8^ University of Toronto Toronto Ontario Canada; ^9^ Institut national de santé publique du Québec Montréal Quebec Canada; ^10^ Community Based Research Centre Vancouver British Columbia Canada; ^11^ Simon Fraser University Burnaby British Columbia Canada

**Keywords:** PrEP awareness, PrEP use, HIV prevention, GBM, temporal trends, health policy

## Abstract

**Introduction:**

Accessibility of pre‐exposure prophylaxis (PrEP) in Canada remains complex as publicly funded coverage and delivery differs by province. In January 2018, PrEP became publicly funded and free of charge in British Columbia (BC), whereas PrEP coverage in Ontario and Montreal is more limited and may require out‐of‐pocket costs. We examined differences over time in PrEP uptake and assessed factors associated with PrEP awareness and use.

**Methods:**

Gay, bisexual and other men who have sex with men (GBM) were recruited through respondent‐driven sampling (RDS) in Toronto, Vancouver and Montreal, Canada, in a prospective biobehavioural cohort study. We applied generalized estimating equations with hierarchical data (RDS chain, participant, visit) to examine temporal trends of PrEP use and correlates of PrEP awareness and use from 2017 to 2020 among self‐reported HIV‐negative/unknown GBM.

**Results:**

Of 2008 self‐identified HIV‐negative/unknown GBM at baseline, 5093 study visits were completed from February 2017 to March 2020. At baseline, overall PrEP awareness was 88% and overall PrEP use was 22.5%. During our study period, we found PrEP use increased in all cities (all *p*<0.001): Montreal 14.2% during the first time period to 39.3% during the last time period (*p*<0.001), Toronto 21.4–31.4% (*p*<0.001) and Vancouver 21.7–59.5% (*p*<0.001). Across the study period, more Vancouver GBM used PrEP than Montreal GBM (aOR = 2.05, 95% CI = 1.60–2.63), with no significant difference between Toronto and Montreal GBM (aOR = 0.90, 95% CI = 0.68–1.18).

**Conclusions:**

Full free‐of‐charge public funding for PrEP in BC likely contributed to differences in PrEP awareness and use. Increasing public funding for PrEP will improve accessibility and uptake among GBM most at risk of HIV.

## 1 INTRODUCTION

In 2020, gay, bisexual and other men who have sex with men (GBM) accounted for 60.8% of new HIV diagnoses in Canada, the highest population proportion, despite representing only 3–4% of the population [1]. Ontario, Quebec and British Columbia (BC) represent three of four Canadian provinces with the highest number and proportion of HIV, which are concentrated within metropolitan areas [2, 3, 4]. HIV pre‐exposure prophylaxis (PrEP) is an effective antiretroviral medication shown to reduce HIV acquisition by 86% in clinical trials [5, 6]. In February 2016, tenofovir disoproxil fumarate/emtricitabine was granted market authorization as PrEP in Canada [7], with three generic options further approved in 2017 [8].

Considering healthcare in Canada is distributed provincially, coverage of PrEP remains complex with different policies between provinces [9]. Nationally, very specific groups (e.g. Canadian Armed Forces, the Inuit and First Nations people) have full PrEP coverage under federal programmes free of charge to the consumers [10]. BC has offered publicly funded free of charge PrEP since January 2018 for those eligible [11]. In Ontario and Montreal, provincial PrEP coverage is more limited [12]. Quebec has provided continuous public funding since 2013 through a tiered payment plan offering full coverage for those <18 years old, full‐time students <25 without a spouse or living with parents and individuals with functional impairment or on social assistance, others will require a maximum payment of $93.08/month ($1117.00 per year) [12]. Ontario has a patchwork of coverage. Full coverage is offered under The Ontario Health Insurance Plan for those **≤**24 and the Ontario Drug Benefit for those ≥65 qualifying with Ontario Disability Support Programme or Ontario Works. Partial coverage is available for those qualifying under Trillium (a programme for Ontario residents who spend 4% or more of after‐tax household income on prescription‐drug cost), with annual deductibles up to $3100 scaled to household income, for those with no insurance, PrEP can cost $250 per month [12]. Although Canada has varying levels of free/low‐cost options, affordability of PrEP remains a key barrier to PrEP uptake internationally for GBM [13].

Prior to PrEP approval, a 2015 online cross‐sectional survey of Canadian GBM found that 54.7% were aware of PrEP and 47.7% were interested in using PrEP, but use was not reported [14]. After approval, a 2017 national sample of 6059 GBM found that 8.4% had ever used PrEP and 86.4% were aware of PrEP [15]. Canadian pharmacology data found an almost fivefold increase in PrEP use among males between 2016 and 2020 [16]. PrEP data released by the BC Centre for Excellence in HIV/AIDS found PrEP use increased from 2177 users in Q3 2018 to 3572 users in Q4 2020 [17, 18]. In Ontario, a sixfold increase in PrEP use was found between 2016 and 2020 from 128.5 per 1,000,000 to 760.5 per 1,000,000 [16] with increases attributed to generic PrEP approval and partial provincial public funding in 2018 [8]. In Quebec, PrEP use has increased almost three and a half times from 196.2 per 1,00,000 in 2016 to 675.2 per 1,000,000 in 2020 [16].

Given the evolutions in PrEP policy and access, we sought to explore how PrEP use has changed over time among self‐reported HIV‐negative/unknown GBM in Canada's three largest cities: Toronto, Montreal and Vancouver. We examined within and between city trends from 2017 to 2020 and we assessed factors associated with PrEP awareness and use among a pooled sample of these urban GBM.

## 2 METHODS

### 2.1 Procedures

Data come from the Engage Study, a longitudinal biobehavioural cohort study of GBM in Toronto, Montreal and Vancouver [2, 19]. Baseline data were collected from February 2017 to August 2019 and participants were recruited using respondent‐driven sampling (RDS) [20]. Our RDS protocol is described in more detail elsewhere [21]. Study visits occurred every 6 months in Vancouver. In Toronto and Montreal, study visits occurred every 12 months for the first 2 years, and thereafter every 6 months. All participants signed an informed consent form prior to data collection. The study was approved by research ethics boards at Ryerson University, University of Toronto, St. Michael's Hospital, University of Windsor, University of British Columbia, Providence Health Care, University of Victoria, Simon Fraser University and the Research Institute of the McGill University Health Centre.

### 2.2 Outcome measures

PrEP awareness was assessed (after giving the definition of PrEP), “Before today, had you ever heard of PrEP?” Responses were either Yes or No. Lifetime PrEP use (including current and former use, and both daily and on‐demand regimens) was assessed by asking, “Have you ever taken PrEP yourself?” Responses were either Yes or No.

### 2.3 Explanatory measures

Explanatory measures included healthcare access, sexual risk factors, sexually transmitted infections (STI) diagnoses and socio‐demographics. Relationship questions included open relationship status and whether participants knew the HIV status of their main partner. We asked participants if they had a primary healthcare provider (PCP), if they were out about their sexuality with their PCP and if they had insurance coverage for prescription medication. We included the HIRI‐MSM scale, a clinical tool used to measure HIV risk, with dichotomized scores of ≥10 indicating high HIV risk and good candidates for PrEP [11, 22]. We assessed participants’ current self‐perceived risk of acquiring HIV, categorized into (very) unlikely; somewhat likely; and (very) likely with “I think I already have HIV.” We also asked participants if they had ever been diagnosed with an STI and if they used crystal methamphetamine or GHB (substances often used in chemsex/Party and Play) in the past 6 months. We created a derived variable on the presence or absence of any condomless anal sex (CAS) with an HIV‐positive or unknown serostatus partner in the past 6 months. Lastly, we assessed the number of male sex partners in the past 6 months.

### 2.4 Analyses

We applied the Wald chi‐square test to examine differences in descriptive results, including city‐specific counts and percentages, and RDS‐II adjusted percentages [23]. RDS‐II sampling weights accounted for network size defined as the number of eligible GBM the participant knew who lived or worked in their city. These are inverse probability sampling weights that are proportional to the participants’ network size [24]. Trend analyses include overall and city‐specific counts and percentages from February 2017 to March 2020, measured in 6‐month increments; we included data collection up until COVID‐19 restrictions were implemented in Canada. We used univariable generalized estimating equations (GEE) with logistic regression to assess significant differences across cities and to assess trends within each city over time. To identify correlates of PrEP awareness and lifetime use, we first applied univariable GEE logistic regression with data pooled across cities and calculated odds ratios (OR) and associated *p*‐values. Variables that were statistically significant at *p<*0.2 and theoretically relevant to our analysis were included for consideration in our final model using a backward stepwise selection approach and we used Quasi Information Criterion (QIC) to compare model fit [25]. The final multivariable model reports adjusted odds ratios (aOR) with 95% confidence intervals (CI). Significance was assessed based on *p*<0.05. We used three‐level hierarchical data (RDS chain, participant‐level, visit‐level) for all regression analyses and included the city as a covariate. All analyses were performed using SAS version 9.4 (SAS, Cary, North Carolina, USA). A planned post‐hoc sensitivity analysis was undertaken to assess if results would differ for PrEP‐eligible GBM, defined as reporting CAS and ANY of the following (1. infectious syphilis or rectal bacterial STI, particularly in the past 12 months; 2. use of non‐occupational post‐exposure prophylaxis more than once; 3. ongoing relationship with an HIV‐positive partner without an undetectable viral load; and 4. HIRI‐MSM score ≥10) [11]. Using baseline data, we include a PrEP‐to‐need ratio defined as the percentage of participants who ever used PrEP among those who are PrEP eligible in each city. We also conducted a lost‐to‐follow‐up analysis comparing participants’ baseline HIRI score ≥10 and whether they had at least one follow‐up visit.

## 3 RESULTS

Our analysis included 2008 self‐reported HIV‐negative/unknown participants, 968 (48.2%) in Montreal, 418 (20.8%) in Toronto and 622 (31.0%) in Vancouver recruited from February 2017 to August 2019. At enrolment, we found that overall 88% (*n* = 1768) of participants had ever heard of PrEP, with RDS‐adjusted estimates indicating significant differences in awareness between Montreal (71.7%), Toronto (82.4%) and Vancouver (92.9%) (*p*<0.001). Overall, 22.5% (*n* = 398) of participants reported lifetime PrEP use at enrolment; RDS‐adjusted estimates from between city analysis indicated significant differences in lifetime PrEP use between Montreal (13.1%), Toronto (14.1%) and Vancouver (20.3%) (*p* = 0.002). PrEP eligibility varied between Montreal (45.8%), Toronto (43.9%) and Vancouver (57.1%) (*p*<0.001). Full descriptive results are in Table 1.

**Table 1:** Crude and respondent‐driven sampling‐adjusted estimates of socio‐demographic and PrEP outcome measures among GBM in Montreal, Toronto and Vancouver

	Overall (*N* = 2008)		Montreal (*N* = 968)				Toronto (*N* = 418)		Vancouver (*N* = 622)			
	Crude		RDS adjusted				RDS adjusted		RDS adjusted			
	*N*	(%)	(%)	(95% CI)		(%)	(95% CI)		(%)	(95% CI)		Adjusted *p*‐value^a^
Age at interview^b^												**<0.001**
<30	858	(42.7)	(41.2)	(35.6)	(46.8)	(57.6)	(48.6)	(66.6)	(53.9)	(46.3)	(61.6)	
30–44	783	(39.0)	(37.2)	(31.3)	(43.2)	(28.2)	(21.4)	(35.0)	(30.8)	(23.9)	(37.6)	
≥45	367	(18.3)	(21.6)	(16.3)	(26.9)	(14.2)	(5.7)	(22.7)	(15.3)	(9.5)	(21.1)	
Annual income^c^												**<0.001**
<$30,000	986	(49.1)	(66.4)	(61.3)	(71.6)	(60.0)	(51.1)	(69.0)	(55.6)	(48.1)	(63.0)	
$30,000–$59,999	635	(31.6)	(25.2)	(20.5)	(30.0)	(27.4)	(18.7)	(36.1)	(29.3)	(22.3)	(36.3)	
≥$60,000	387	(19.3)	(8.3)	(5.8)	(10.8)	(12.5)	(8.1)	(17.0)	(15.2)	(11.3)	(19.0)	
Ethnicity												**<0.001**
Canadian	1007	(50.1)	(52.2)	(46.3)	(58.2)	(31.2)	(22.5)	(39.9)	(39.5)	(31.9)	(47.2)	
Aboriginal	23	(1.1)	(1.3)	(0.0)	(3.0)	(2.8)	(0.0)	(6.8)	(1.7)	(0.2)	(3.1)	
European	365	(18.2)	(15.4)	(11.2)	(19.7)	(25.9)	(17.0)	(34.8)	(14.8)	(10.4)	(19.1)	
Asian	223	(11.1)	(5.3)	(2.8)	(7.9)	(13.9)	(8.7)	(19.0)	(24.5)	(17.9)	(31.2)	
African/Caribbean/Black	53	(2.6)	(2.9)	(1.3)	(4.5)	(7.5)	(2.4)	(12.7)	(0.7)	(0.1)	(1.3)	
Mixed race/ethnicity	55	(2.7)	(2.0)	(0.9)	(3.1)	(4.2)	(1.0)	(7.5)	(2.8)	(0.1)	(5.5)	
Other	282	(14.0)	(20.8)	(15.5)	(26.0)	(14.5)	(9.1)	(19.9)	(16.0)	(9.8)	(22.2)	
Sexual identity												**<0.001**
Gay	1613	(80.3)	(74.1)	(68.7)	(79.5)	(71.7)	(63.5)	(79.9)	(81.6)	(75.3)	(87.9)	
Bisexual	138	(6.9)	(13.4)	(9.2)	(17.6)	(10.9)	(4.5)	(17.3)	(8.9)	(4.8)	(13.1)	
Other	257	(12.8)	(12.5)	(8.3)	(16.7)	(17.4)	(11.1)	(23.8)	(9.5)	(4.2)	(14.7)	
Education completed												**<0.001**
**≤**High school	296	(14.7)	(24.3)	(18.8)	(29.8)	(17.9)	(9.3)	(26.5)	(14.7)	(9.5)	(19.8)	
>High school	1712	(85.3)	(75.7)	(70.2)	(81.2)	(82.1)	(73.5)	(90.7)	(85.3)	(80.2)	(90.5)	
Gender identity												**<0.001**
Cisgender	1863	(92.8)	(87.7)	(83.0)	(92.5)	(88.1)	(81.8)	(94.4)	(94.8)	(91.7)	(97.9)	
Another gender identity	145	(7.2)	(12.3)	(7.5)	(17.0)	(11.9)	(5.6)	(18.2)	(5.2)	(2.1)	(8.3)	
Ever heard of PrEP												**<0.001**
No	240	(12.0)	(28.3)	(22.0)	(34.6)	(17.6)	(10.0)	(25.3)	(17.4)	(10.6)	(24.2)	
Yes	1768	(88.0)	(71.7)	(65.4)	(78.0)	(82.4)	(74.7)	(90.0)	(82.6)	(75.8)	(89.4)	
PrEP eligible												**<0.001**
No	826	(42.3)	(54.2)	(48.2)	(60.2)	(56.1)	(47.2)	(65.0)	(42.9)	(35.3)	(50.5)	
Yes	1128	(57.7)	(45.8)	(39.8)	(51.8)	(43.9)	(35.0)	(52.8)	(57.1)	(49.5)	(64.7)	
Ever taken PrEP^d^												**0.002**
No	1370	(77.5)	(86.9)	(83.1)	(90.7)	(85.9)	(81.3)	(90.6)	(79.7)	(74.3)	(85.1)	
Yes	398	(22.5)	(13.1)	(9.3)	(16.9)	(14.1)	(9.4)	(18.7)	(20.3)	(14.9)	(25.7)	
PrEP regimen^e^												**0.001**
Daily/continuously	305	(76.6)	(61.6)	(46.1)	(77.1)	(87.3)	(75.9)	(98.7)	(78.8)	(65.0)	(92.5)	
On demand	41	(10.3)	(10.5)	(4.2)	(16.9)	(5.5)	(0.0)	(14.7)	(13.8)	(0.4)	(27.3)	
Both ways	52	(13.1)	(27.9)	(12.1)	(43.7)	(7.2)	(0.0)	(14.7)	(7.4)	(1.2)	(13.6)	

^a^ Bold text indicates statistically significant difference with a *p*‐value less than 0.05.

^b^ Age at interview measured in years.

^c^ Annual income measured in Canadian dollars.

^d^ Ever taken PrEP (among those who had heard of PrEP) had a sample size of (*n* = 1768).

^e^ Among participants who have ever taken PrEP (*n* = 398).

### 3.1 Trends in PrEP use

**Figure 1:**
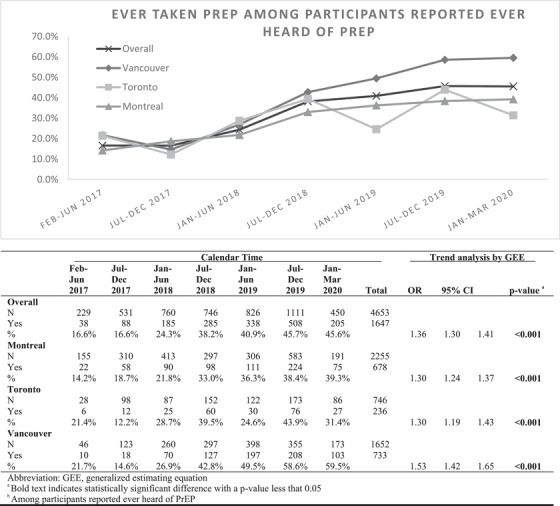
Trends of the percentage who ever took PrEP among those who had heard of PrEP in Montreal, Toronto and Vancouver (February 2017–March 2020).

Of *N* = 5093 study visits by 2008 participants, 1494 (74.4%) participants completed at least one follow‐up visit. The median number of follow‐up visits was 2 (Q1–Q3: 1–3) and the median follow‐up time was 1.7 years (Q1–Q3: 1.1–2.1). Overall, lifetime PrEP use increased during our study period (February–June 2017: 16.6%, January–March 2020: 45.6%, *p*<0.001). Further, we found PrEP use increased in all three cities. From first time period to last, Montreal increased from 14.2% to 39.3% (*p*<0.001), Toronto 21.4% to 31.4% (*p*<0.001) and Vancouver 21.7% to 59.5% (*p*<0.001). Full results are in Figure 1.

### 3.2 Multivariable results for PrEP awareness and use

Participants in Vancouver (aOR = 2.01, 95% CI = 1.34–3.01) were more aware of PrEP than those in Montreal, we found no significant difference between Toronto (aOR = 1.50, 95% CI = 0.92–2.44) and Montreal. Furthermore, participants who reported being in a (partially) open relationship versus closed/monogamous relationship (aOR = 2.23, 95% CI = 1.25–4.00), had a HIRI‐MSM score ≥10 (aOR = 1.87, 95% CI = 1.29–2.71), had ever been diagnosed with an STI (aOR = 1.66, 95% CI = 1.17–2.24) and reported any CAS with an HIV‐positive or unknown status partner in the past 6 months (aOR = 1.87, 95% CI = 1.27–2.77) were more likely to be aware of PrEP. Having prescription medication insurance was positively associated with PrEP awareness (aOR = 1.62, 95% CI = 1.17–2.24). Full univariable and multivariable results are in Table 2.

**Table 2:** Univariable and multivariable generalized estimating equations assessing correlates of ever having heard of PrEP among GBM in Montreal, Toronto and Vancouver

	Ever heard of PrEP										Univariable GEE				Multivariable GEE			
		No (*n* = 240)				Yes (*n* = 1768)					Ever heard of PrEP Yes versus No				Ever heard of PrEP Yes versus No			
Variable	Total *N*	*N*	(RDS %)	(95% CI)		*N*	(RDS %)	(95% CI)		*p*‐value^a^	OR	95% CI		*p*‐value	aOR	95% CI		*p*‐value
City	2008									**<0.001**								
Montreal		166	(60.1)	(49.6)	(70.6)	802	(44.7)	(40.4)	(49.0)									
Toronto		30	(16.2)	(8.8)	(23.5)	388	(22.2)	(18.4)	(26.0)		2.74	1.78	4.21	**<0.001**	1.50	0.92	2.44	0.100
Vancouver		44	(23.7)	(14.5)	(33.0)	578	(33.1)	(28.9)	(37.4)		3.07	2.13	4.44	**<0.001**	2.01	1.34	3.01	**0.001**
Age at interview^b^	2008									**<0.001**								
<30		70	(32.3)	(22.7)	(42.0)	788	(53.3)	(48.9)	(57.7)									
30–44		64	(26.3)	(16.9)	(35.6)	719	(35.4)	(31.2)	(39.6)		1.05	0.76	1.45	0.748	0.55	0.36	0.85	**0.006**
≥45		106	(41.4)	(30.4)	(52.4)	261	(11.3)	(8.7)	(13.9)		0.26	0.18	0.36	**<0.001**	0.12	0.08	0.18	**<0.001**
Annual income^c^	2008									**<0.001**								
Less than $30,000		173	(74.1)	(64.2)	(84.1)	813	(58.1)	(53.9)	(62.2)									
$30,000–$59,999		56	(21.7)	(12.0)	(31.3)	579	(28.5)	(24.7)	(32.3)		2.03	1.61	2.56	**<0.001**	1.55	1.10	2.19	**0.012**
$60,000 and over		11	(4.2)	(0.4)	(8.0)	376	(13.4)	(11.2)	(15.6)		5.38	3.49	8.30	**<0.001**	4.11	2.04	8.29	**<0.001**
Ethnicity	2008									**<0.001**								
Canadian		145	(53.3)	(42.5)	(64.1)	862	(41.2)	(36.9)	(45.5)									
Aboriginal		2	(1.8)	(0.0)	(5.3)	21	(1.7)	(0.4)	(2.9)		1.60	0.31	8.24	0.575				
European		31	(12.3)	(4.8)	(19.8)	334	(18.9)	(15.5)	(22.3)		1.47	1.01	2.14	**0.043**				
Asian		23	(12.2)	(6.0)	(18.3)	200	(13.3)	(10.3)	(16.3)		0.80	0.52	1.25	0.332				
African/Caribbean/Black		6	(5.0)	(0.4)	(9.6)	47	(2.7)	(1.6)	(3.8)		1.60	0.65	3.95	0.309				
Mixed race/ethnicity		7	(1.6)	(0.0)	(3.1)	48	(3.1)	(1.6)	(4.5)		1.16	0.54	2.48	0.709				
Other		26	(13.9)	(5.8)	(22.0)	256	(19.2)	(15.5)	(22.9)		1.30	0.91	1.84	**0.148**				
Sexual identity	2008									**<0.001**								
Gay		141	(59.8)	(49.3)	(70.3)	1472	(80.7)	(77.1)	(84.2)									
Bisexual		67	(27.3)	(18.4)	(36.2)	71	(6.8)	(4.5)	(9.2)		0.20	0.14	0.28	**<0.001**	0.35	0.22	0.55	**<0.001**
Another sexual identity		32	(12.9)	(4.9)	(20.9)	225	(12.5)	(9.6)	(15.5)		0.83	0.58	1.18	0.302	1.10	0.62	1.94	0.754
Education completed	2008									**<0.001**								
High school or less		98	(40.4)	(29.9)	(51.0)	198	(14.0)	(10.7)	(17.3)									
Greater than high school		142	(59.6)	(49.0)	(70.1)	1570	(86.0)	(82.7)	(89.3)		4.32	3.34	5.58	**<0.001**	2.79	1.97	3.94	**<0.001**
Gender identity	2008									**0.005**								
Cisgender		203	(86.5)	(79.3)	(93.6)	1660	(91.0)	(88.0)	(94.1)									
Another gender identity		37	(13.5)	(6.4)	(20.7)	108	(9.0)	(5.9)	(12.0)		0.59	0.42	0.81	**0.001**	0.44	0.26	0.75	**0.003**
Know the HIV status of the main partner	2008									**<0.001**								
No		19	(4.8)	(1.4)	(8.1)	28	(1.8)	(1.0)	(2.6)									
Yes (certain/think HIV negative)		69	(29.8)	(20.0)	(39.6)	735	(44.7)	(40.3)	(49.1)		4.50	2.63	7.71	**<0.001**	3.71	1.68	8.21	**0.001**
Yes (certain/think HIV positive)		9	(3.7)	(0.0)	(7.5)	63	(3.0)	(1.7)	(4.3)		4.09	2.06	8.13	**<0.001**	1.93	0.68	5.47	0.215
No main partner		143	(61.8)	(51.5)	(72.1)	942	(50.5)	(46.1)	(54.9)		3.36	2.01	5.61	**<0.001**	NA			
Open relationship^d^	2008									**<0.001**								
Agreed to only have sex with each other		24	(10.1)	(4.3)	(15.9)	170	(14.5)	(10.7)	(18.2)									
No discussion/no agreement		37	(16.6)	(8.0)	(25.2)	165	(12.5)	(9.4)	(15.5)		0.61	0.42	0.89	**0.010**	1.10	0.61	1.93	0.745
Open/partially open		36	(11.5)	(5.9)	(17.1)	491	(22.6)	(19.3)	(25.9)		1.75	1.20	2.55	**0.004**	2.37	1.30	4.32	**0.005**
No main partner		143	(61.8)	(51.5)	(72.1)	942	(50.5)	(46.1)	(54.9)		0.96	0.70	1.33	0.819	NA			
Out to the current regular primary healthcare provider	1886									**<0.001**								
No		26	(18.6)	(8.8)	(28.4)	138	(11.9)	(8.7)	(15.0)									
Yes		87	(30.1)	(20.6)	(39.6)	952	(44.1)	(39.8)	(48.5)		2.35	1.65	3.34	**<0.001**	1.60	0.94	2.73	0.085
No regular primary healthcare provider		102	(51.3)	(40.1)	(62.5)	581	(44.0)	(39.5)	(48.5)		1.35	0.94	1.93	**0.102**	0.93	0.52	1.65	0.797
Insurance for prescription medicine	2008									**<0.001**								
No		105	(46.1)	(35.4)	(56.8)	503	(34.8)	(30.5)	(39.2)									
Yes		135	(53.9)	(43.2)	(64.6)	1265	(65.2)	(60.8)	(69.5)		2.04	1.63	2.56	<0.001	1.62	1.17	2.24	0.004
HIRI score > = 10	1927									**<0.001**								
No		141	(67.0)	(56.5)	(77.5)	515	(36.2)	(31.9)	(40.5)									
Yes		79	(33.0)	(22.5)	(43.5)	1192	(63.8)	(59.5)	(68.1)		3.13	2.45	4.01	**<0.001**	1.87	1.29	2.71	**0.001**
Current risk of getting HIV	1961									**<0.001**								
Very unlikely/unlikely		167	(73.9)	(64.4)	(83.4)	1406	(81.8)	(78.9)	(84.7)									
Somewhat likely		30	(14.4)	(7.4)	(21.5)	227	(13.0)	(10.5)	(15.4)		0.84	0.60	1.17	0.311	0.60	0.36	1.01	0.055
Very likely/likely/think already have HIV		22	(11.6)	(4.1)	(19.1)	109	(5.2)	(3.7)	(6.8)		0.67	0.45	0.99	**0.046**	0.55	0.29	1.05	0.069
Any high‐risk sex (P6M)	1971									**<0.001**								
No		189	(86.4)	(78.3)	(94.5)	1042	(63.4)	(59.2)	(67.6)									
Yes		38	(13.6)	(5.5)	(21.7)	702	(36.6)	(32.4)	(40.8)		2.22	1.75	2.81	**<0.001**	1.87	1.27	2.77	**0.002**
Ever been diagnosed with an STI	1976									**<0.001**								
No		131	(61.8)	(51.0)	(72.6)	692	(50.0)	(45.6)	(54.4)									
Yes		100	(38.2)	(27.4)	(49.0)	1053	(50.0)	(45.6)	(54.4)		2.01	1.57	2.57	**<0.001**	1.66	1.17	2.34	**0.004**
Any chemsex drugs (P6M)																		
No	1967	194	(86.2)	(78.7)	(93.8)	1463	(88.1)	(85.0)	(91.3)	0.292								
Yes		32	(13.8)	(6.2)	(21.3)	278	(11.9)	(8.7)	(15.0)		1.31	0.95	1.79	0.096	0.94	0.57	1.56	0.821

	Total *N*	Median	Adjusted median	Adjusted Q1	Adjusted Q3	Median	Adjusted median	Adjusted Q1	Adjusted Q3	Adjusted *p*‐value	OR	95% CI		*p*‐value	aOR	95% CI		*p*‐value
Number of male sex partners (P6M)	2008	3	2	1	5	6	5	2	10	**<0.001**	1.06	1.02	1.09	**0.001**	1.03	1.00	1.05	**0.042**

Note: Analyses controlled for city in regressions.

Abbreviations: GEE, generalized estimating equations; PCP, primary healthcare provider; P6M, past 6 months.

^a^ Bold text indicates statistically significant difference with a *p*‐value less than 0.05.

^b^ Age at interview measured in years.

^c^ Annual income measured in Canadian dollars.

^d^ Removed at visit 4 in Montreal and Toronto and removed on 11 January 2020 in Vancouver.

Lifetime PrEP use was more likely among participants in Vancouver (aOR = 2.21, 95% CI = 1.72–2.84) than in Montreal, with no significant difference between Toronto and Montreal GBM (aOR = 1.01, 95% CI = 0.73–1.33). Lifetime PrEP use was more likely among those who were certain or thought their main partner was HIV positive (aOR = 3.27, 95% CI = 1.12–9.49), compared to those who did not know the HIV status of their main partner. GBM were more likely to have ever used PrEP if they had a HIRI‐MSM score ≥10 (aOR = 1.95, 95% CI = 1.60–2.38), reported any CAS with an HIV‐positive or unknown status partner in the past 6 months (aOR = 2.13, 95% CI = 1.81–2.51) and had ever been diagnosed with an STI (aOR = 2.37, 95% CI = 1.89–2.97). Moreover, PrEP use was positively associated with insurance coverage for prescription medication (aOR = 1.35, 95% CI = 1.11–1.64) and use of chemsex drugs in the past 6 months (aOR = 1.69, 95% CI = 1.34–2.12). GBM who self‐reported being “somewhat likely” to acquire HIV (aOR = 0.45, 95% CI = 0.33–0.60) or as “(very) likely”/“I think already have HIV” (aOR = 0.54, 95% CI = 0.37–0.77) were less likely to have ever taken PrEP in comparison with those who self‐reported they were “(very) unlikely” to acquire HIV. Full univariable and multivariable results are in Table 3.

**Table 3:** Univariable and multivariable generalized estimating equations assessing correlates of reporting ever having taken PrEP among GBM who have ever heard of PrEP in Montreal, Toronto and Vancouver

		Ever taken PrEP									Univariable GEE				Multivariable GEE			
		No (*n* = 1370)				Yes (*n* = 398)					Ever taken PrEP Yes versus No				Ever taken PrEP Yes versus No			
Variable	Total *N*	*N*	(RDS %)	(95% CI of RDS %)		*N*	(RDS %)	(95% CI of RDS %)		*p*‐value^a^	OR	95% CI		*p*‐value	aOR	95% CI		*p*‐value
City	1768									**0.002**								
Montreal		657	(46.1)	(41.2)	(50.9)	145	(37.3)	(28.6)	(46.0)									
Toronto		294	(22.6)	(18.2)	(27.0)	94	(19.9)	(14.0)	(25.9)		1.15	0.89	1.49	0.283	1.01	0.76	1.33	0.961
Vancouver		419	(31.3)	(26.5)	(36.1)	159	(42.8)	(34.1)	(51.5)		1.93	1.57	2.38	**<0.001**	2.21	1.72	2.84	**<0.001**
Age at interview^b^	1768									**<0.001**								
<30		658	(55.5)	(50.5)	(60.4)	130	(41.8)	(32.7)	(50.9)									
30–44		517	(33.7)	(28.9)	(38.5)	202	(44.7)	(36.2)	(53.2)		1.85	1.53	2.23	**<0.001**	1.28	1.02	1.60	**0.034**
≥45		195	(10.9)	(7.9)	(13.8)	66	(13.5)	(8.4)	(18.5)		1.43	1.06	1.93	**0.020**	0.93	0.68	1.29	0.678
Annual income^c^	1768									**0.007**								
Less than $30,000		655	(59.3)	(54.6)	(63.9)	158	(51.7)	(43.0)	(60.4)									
$30,000–$59,999		457	(28.5)	(24.2)	(32.7)	122	(28.7)	(20.7)	(36.7)		1.41	1.20	1.65	**<0.001**	1.30	1.08	1.57	**0.006**
$60,000 and over		258	(12.3)	(9.9)	(14.6)	118	(19.6)	(14.3)	(24.8)		1.82	1.46	2.28	**<0.001**	1.28	0.97	1.67	0.076
Ethnicity	1768									**0.078**								
Canadian		672	(41.2)	(36.4)	(46.0)	190	(41.1)	(32.9)	(49.3)									
Aboriginal		18	(1.4)	(0.4)	(2.3)	3	(3.5)	(0.0)	(9.7)		0.81	0.35	1.88	0.625	0.77	0.28	2.13	0.610
European		254	(18.6)	(14.9)	(22.4)	80	(20.3)	(12.9)	(27.8)		1.24	0.97	1.59	**0.089**	1.31	1.00	1.71	0.054
Asian		147	(13.0)	(9.6)	(16.3)	53	(15.2)	(9.0)	(21.3)		1.32	0.97	1.81	**0.080**	1.97	1.42	2.74	**<0.001**
African/Caribbean/Black		39	(2.7)	(1.5)	(4.0)	8	(2.3)	(0.2)	(4.4)		1.12	0.66	1.89	0.680	1.13	0.54	2.36	0.751
Mixed race/ethnicity		41	(3.5)	(1.8)	(5.3)	7	(0.6)	(0.1)	(1.2)		1.05	0.59	1.86	0.876	1.23	0.69	2.19	0.475
Other		199	(19.6)	(15.4)	(23.8)	57	(17.0)	(10.2)	(23.7)		1.09	0.79	1.50	0.613	1.53	1.11	2.12	**0.010**
Sexual identity	1768									**0.006**								
Gay		1122	(79.2)	(75.1)	(83.3)	350	(88.2)	(83.4)	(93.1)									
Bisexual		59	(7.3)	(4.6)	(10.0)	12	(4.3)	(0.6)	(8.1)		0.74	0.51	1.08	**0.115**				
Other		189	(13.5)	(10.0)	(16.9)	36	(7.4)	(4.1)	(10.8)		0.95	0.76	1.19	0.648				
Education	1768									**<0.001**								
High school or less		164	(15.5)	(11.6)	(19.4)	34	(6.1)	(3.3)	(8.9)									
Greater than high school		1206	(84.5)	(80.6)	(88.4)	364	(93.9)	(91.1)	(96.7)		1.42	1.12	1.80	**0.004**	1.29	0.95	1.75	0.103
Gender identity	1768									**0.016**								
Cisgender		1279	(90.2)	(86.7)	(93.8)	381	(95.2)	(91.7)	(98.6)									
Another gender identity		91	(9.8)	(6.2)	(13.3)	17	(4.8)	(1.4)	(8.3)		1.09	0.83	1.42	0.532				
Know the HIV status of the main partner	1768									**<0.001**								
No		27	(2.1)	(1.1)	(3.1)	1	(0.1)	(0.0)	(0.4)									
Yes (certain/think HIV negative)		591	(47.7)	(42.7)	(52.7)	144	(28.8)	(22.0)	(35.7)		3.30	1.56	6.97	**0.002**	2.11	0.78	5.70	0.139
Yes (certain/think HIV positive)		33	(2.1)	(0.9)	(3.3)	30	(7.8)	(2.7)	(12.8)		7.03	2.98	16.54	**<0.001**	3.27	1.12	9.49	**0.030**
No main partner		719	(48.1)	(43.2)	(53.0)	223	(63.2)	(55.3)	(71.1)		3.81	1.80	8.08	**0.001**	NA			
Open relationship^d^	1768									**<0.001**								
Agreed to only have sex with each other		154	(16.6)	(12.3)	(20.9)	16	(3.0)	(1.0)	(4.9)									
No discussion/no agreement		140	(13.4)	(9.9)	(16.9)	25	(7.6)	(3.4)	(11.8)		1.16	0.88	1.52	0.301	1.09	0.78	1.51	0.619
Open/partially open		357	(22.0)	(18.2)	(25.7)	134	(26.2)	(19.3)	(33.1)		1.51	1.19	1.91	**0.001**	1.27	0.96	1.67	0.095
No main partner		719	(48.1)	(43.2)	(53.0)	223	(63.2)	(55.3)	(71.1)		1.48	1.19	1.84	**0.001**	NA			
Out to the current regular primary healthcare provider	1671									**<0.001**								
No		125	(12.9)	(9.2)	(16.5)	13	(6.9)	(1.4)	(12.4)									
Yes		653	(39.9)	(35.0)	(44.8)	299	(65.2)	(55.8)	(74.6)		3.28	2.33	4.61	**<0.001**	3.56	2.37	5.36	**<0.001**
No regular primary healthcare provider		502	(47.2)	(42.2)	(52.3)	79	(27.9)	(18.8)	(37.0)		1.25	0.90	1.75	**0.185**	1.39	0.92	2.11	0.119
Insurance for prescription medicine	1768									**<0.001**								
No		428	(37.8)	(32.9)	(42.7)	75	(18.8)	(12.2)	(25.4)									
Yes		942	(62.2)	(57.3)	(67.1)	323	(81.2)	(74.6)	(87.8)		1.48	1.27	1.74	**<0.001**	1.35	1.11	1.64	**0.002**
HIRI score > = 10	1707									**<0.001**								
No		481	(40.8)	(35.9)	(45.6)	34	(12.3)	(6.2)	(18.4)									
Yes		842	(59.2)	(54.4)	(64.1)	350	(87.7)	(81.6)	(93.8)		2.29	1.97	2.67	**<0.001**	1.95	1.60	2.38	**<0.001**
Current risk of getting HIV	1742									**0.060**								
Very unlikely/unlikely		1087	(82.0)	(78.9)	(85.2)	319	(80.6)	(73.7)	(87.5)									
Somewhat likely		187	(13.3)	(10.6)	(16.1)	40	(11.2)	(5.8)	(16.6)		0.63	0.50	0.80	**<0.001**	0.45	0.33	0.61	**<0.001**
Very likely/likely/think already have HIV		76	(4.7)	(3.1)	(6.3)	33	(8.2)	(3.4)	(13.1)		0.73	0.53	1.02	**0.065**	0.54	0.37	0.77	**0.001**
Any high‐risk sex (P6M)	1744									**<0.001**								
No		927	(68.9)	(64.2)	(73.6)	115	(34.9)	(26.3)	(43.5)									
Yes		420	(31.1)	(26.4)	(35.8)	282	(65.1)	(56.5)	(73.7)		2.56	2.24	2.92	**<0.001**	2.13	1.81	2.51	**<0.001**
Ever been diagnosed with any STI	1745									**<0.001**								
No		621	(54.4)	(49.4)	(59.4)	71	(26.5)	(18.0)	(35.1)									
Yes		729	(45.6)	(40.6)	(50.6)	324	(73.5)	(64.9)	(82.0)		3.46	2.82	4.25	**<0.001**	2.37	1.89	2.97	**<0.001**
Any chemsex drugs (P6M)	1741									**<0.001**								
No		1184	(89.9)	(86.3)	(93.4)	279	(79.0)	(72.1)	(85.8)									
Yes		168	(10.1)	(6.6)	(13.7)	110	(21.0)	(14.2)	(27.9)		2.01	1.67	2.41	<0.001	1.69	1.34	2.12	<0.001

	Total *N*	Median	Adjusted median	Adjusted Q1	Adjusted Q3	Median	Adjusted median	Adjusted Q1	Adjusted Q3	Adjusted *p*‐value	OR	95% CI		*p*‐value	aOR	95% CI		*p*‐value
Number of male sex partners (P6M)	1768	5	4	2	8	15	10	6	25	**<0.001**	1.02	1.01	1.03	**<0.001**	1.01	1.006	1.02	**<0.001**

Note: All analyses controlled for city in regressions.

Abbreviations: GEE, generalized estimating equations; PCP, primary healthcare provider; P6M, past 6 months.

^a^ Bold text indicates statistically significant difference with a *p*‐value less than 0.05.

^b^ Age at interview measured in years.

^c^ Annual income measured in Canadian dollars.

^d^ Removed at visit 4 in Montreal and Toronto and removed on 11 January 2020 in Vancouver.

### 3.3 Post‐hoc analyses for PrEP use among GBM who met PrEP clinical eligibility

PrEP‐eligible participants from Vancouver had greater odds of PrEP use compared with GBM from Montreal (aOR = 1.83, 95% CI = 1.41–2.37), while GBM from Toronto had lower odds of PrEP use compared with Montreal (aOR = 0.71, 95% CI = 0.53–0.96). Among socio‐demographic variables, GBM aged 30–44 versus <30 (aOR = 1.57, 95% CI = 1.24–1.99), and aged >45 versus <30 (aOR = 1.85, 95% CI = 1.24–2.76), GBM with an annual income $30,000–$59,999 versus <$30,000 (aOR = 1.41, 95% CI = 1.15–1.73), an annual income >$60,000 versus <$30,000 (aOR = 1.40, 95% CI = 1.05–1.86) and participants who identified as Asian versus Canadian (aOR = 1.93, 95% CI = 1.34–2.80) were more likely to report PrEP use. Additionally, GBM who had (partially) open relationships (aOR = 1.52, 95% CI = 1.14–2.02) and GBM who were single (aOR = 1.67, 95% CI = 1.27–2.20) had greater odds of PrEP use compared to GBM who had a closed/monogamous relationship with a main partner. GBM who indicated they were out about their sexual orientation to their PCP (aOR = 3.51, 95% CI = 2.32–5.32) also had greater odds of PrEP use compared with GBM who were not out. Reporting a greater number of male sexual partners in the past 6 months was associated with greater odds of PrEP use (aOR = 1.02, 95% CI = 1.02–1.03).

Lastly, we conducted a PrEP‐to‐need ratio for each city. In Montreal, 504 participants met PrEP eligibility at baseline, with 25% indicating they had ever taken PrEP. In Toronto, 245 participants met PrEP eligibility at baseline, with 33.47% ever using PrEP. In Vancouver, 403 participants met PrEP eligibility at baseline, with 34.99% ever reporting PrEP. Our lost‐to‐follow‐up analysis did not find significant differences in HIRI scores between participants with at least one follow‐up visit and those without (*p* = 0.728).

## 4 DISCUSSION

Among 2008 self‐reported HIV‐negative/unknown GBM from Vancouver, Toronto and Montreal, we found that overall lifetime PrEP use increased within all three cities between February 2017 and March 2020. Given the lack of differences found between Montreal and Toronto, both jurisdictions without fully funded PrEP access [12], the finding that Vancouver GBM were more likely to be aware and have used PrEP (even after controlling for demographic and individual‐level correlates) strongly supports the positive impact of free‐of‐charge publicly funded PrEP programmes in improving access and uptake of PrEP.

Since its implementation in January 2018, BC has reported over 3500 new PrEP users, of which, approximately 99% are GBM [18]. Thus, the improved access to freely available PrEP seems to have a direct association with increased uptake. BC's free‐of‐charge publicly funded PrEP programme is a first in Canada and aligned with BC's commitment to ending HIV/AIDS. In 1996, BC pioneered treatment‐as‐prevention, focusing on testing and treating HIV to reduce morbidity, mortality and community transmission [26]. Since implementation, HIV incidence rates have decreased in the province and including PrEP in this plan aims to further reduce transmission [27]. Noticeably, BC's PrEP policy was passed by BC's New Democratic Party, a centre‐left learning party whose platform emphazises social programmes. In comparison, Ontario and Quebec are both led by centre‐right learning parties. Evidently, politics and history in funding HIV programmes played a key role in BC's PrEP policy.

However, we advise some caution in our interpretation as it is not possible to state differences in PrEP use were directly related to policy differences between provinces. City‐specific differences, which are difficult to measure, may also account for differences in PrEP uptake including: the organization and delivery of PrEP programmes; the number of PrEP providers; different types of community‐based organizations for GBM; and access to linguistically and culturally appropriate care. In addition to larger structural barriers, such as costs, individual, interpersonal and other structural barriers factor into PrEP use among GBM [28].

Our multivariable model found GBM who were single, those with main partners who were living with HIV and those in open or partially open relationships had greater odds of using PrEP. Moreover, GBM who had a HIRI score ≥10, who engaged in high‐risk sex in the past 6 months and who have ever had an STI diagnosis were significantly associated with PrEP use. These findings are aligned with guidelines for PrEP use among GBM [11] and profiles of PrEP users from similar high‐income countries, such as engaging in high‐risk sex, reporting a greater number of male anal sex partners and having ongoing relationship with main partner who is living with HIV [29, 30]. Additionally, a discrepancy between HIV risk and PrEP use is highlighted in our findings as GBM with higher self‐perceived HIV risk had lower odds of PrEP use compared to GBM with lower self‐perceived risk. This is aligned with research indicating low rates of PrEP use among GBM with greater HIV risk and PrEP non‐use associated with lack of perceived risk, PrEP scepticism, lack of medical provider and lack of medical insurance [2]. These findings suggest nuanced interventions that create pathways for initiating conversations about PrEP and possible PrEP use independent of patients’ self‐perceived risks.

Our post‐hoc further signifies the importance of PrEP coverage specifically for those who are clinically PrEP eligible and most likely to acquire HIV. Overall, these findings are aligned with previous research among Canadian GBM which found lacking medication insurance and being concerned about the cost of PrEP were associated with PrEP non‐use [31]. Vancouver had the greatest PrEP‐to‐need ratio, indicating that PrEP coverage in Vancouver is highest compared to its needs.

Lifetime PrEP use nearly tripled from the start of our observation, (early 2017: 16%) to the end of our study (early 2020: 45%). In 2019, the World Health Organization reported that PrEP use has increased over time with over 600,000 people across 76 countries receiving PrEP at least once, a 70% increase from 2018 [32]. In context with other Western countries where the HIV epidemic is centred around GBM, our findings are in line with research from Australia [33], the United States [34] and the UK [35].

Apart from Canada, countries including Norway, Scotland and South Africa have demonstrated the success of free‐of‐charge PrEP programmes [36]. In 2016, Norway became the first country to publicly fund PrEP at no cost to citizens. Data from 2019 found 1150 people using PrEP in Norway; of these, 821 are on a daily dosing regimen (71.3%), while 330 are on an intermittent or event‐based dosing regimen (28.7%). Of interest is that approximately 98% of all PrEP users in Norway are GBM [37]. Additionally, PrEP became publicly funded and free of charge in Scotland in July 2017, 1872 individuals were prescribed PrEP within the first year, among whom 99% were GBM [38]. Corresponding with PrEP uptake, Scotland has seen a 19.7% drop in HIV incidence among GBM 2 years pre‐PrEP compared with 2 years post‐PrEP coverage. Additionally, HIV incidence fell 43% from 5.13 to 3.25 per 1000 person‐years [39]. High uptake of PrEP in Scotland may explain the large decrease in HIV incidence [38]. However, by the end of 2020, fewer than 15 countries offered some form of publicly funded PrEP and the resulting uptake of PrEP has been slow [40]. Importantly, we do not suggest that simply offering PrEP at no cost to GBM will solve the HIV pandemic in Canada or internationally. As previously stated, there are several other factors that influence PrEP uptake among GBM, including the number of providers willing to prescribe PrEP, the number and support of community‐based organizations and self‐perceived HIV risk. These challenges are highlighted in France, where HIV is also centred around GBM, and despite free PrEP availability, in 2017, only 9% of GBM in the EMIS study reported ever using PrEP. The authors found that eligible GBM not using PrEP were more likely to be younger, students, less open about their sexuality, living in smaller cities and having lower safer sex self‐efficacy [41]. Therefore, a comprehensive approach to increasing PrEP use must reach those in need, increase demand and improve delivery to fully utilize the benefits of PrEP in reducing HIV.

Implementing a free‐of‐charge publicly funded PrEP programme has a multitude of beneficial implications that can also address larger barriers to care. First, PrEP programmes reduce barriers for those most marginalized, including GBM, those with financial barriers, youths and gender‐diverse individuals. Second, although PrEP does not directly protect against STIs, a protocol for PrEP disbursement is regular HIV and STI testing, which can lead to earlier diagnosis, treatment and engaging individuals in regular care [11]. Third, PrEP may be cost‐effective as preventing HIV infections reduce the costs associated with a lifetime of care. In BC, preliminary provincial results indicate a drop in the incidence rate of HIV from 4.0 per 100,000 in 2018 (when the PrEP programme was introduced) to 3.5 per 100,000 in 2019 [42]. Future research should examine the full PrEP cascade, including PrEP continuation/maintenance [43]. This longitudinal research is essential to examine how GBM start, maintain, stop and restart PrEP over time, especially during and throughout the COVID‐19 pandemic [44]. Given the impacts of the COVID‐19 pandemic, and the significant disruptions to HIV prevention and care services, renewing investments in HIV prevention and care is needed now more than ever. Canada invests $26.4 million annually through the HIV and the Hepatitis C Community Action Fund and an additional $7 million annually on the Harm Reduction Fund to help community‐based organizations address HIV [45]. With competing interests associated with COVID‐19, local advocates are pushing the Canadian government to increase funding to support and sustain the fight against HIV [46, 47]. Internationally, the United States has increased HIV funding annually [48], and the United Kingdom recently announced a £23 million investment to end new HIV infections by 2030 [49].

This study was subject to a number of strengths and limitations. First, data from each city are not proportional to the underlying populations within each city. Second, descriptive results found that GBM in Vancouver were more likely to report behaviours that met eligibility criteria for PrEP. However, when limiting our analysis to those who were PrEP eligible in each city, participants in Vancouver were still significantly more likely to report lifetime PrEP use and GBM from Toronto were less likely to have ever used PrEP compared to GBM in Montreal. Third, PrEP measures were based on self‐reported data where misreporting and social desirability bias are possible. Fourth, RDS recruitment is based on social networks and GBM who are not connected with other gay, bisexual, transgender, two‐spirit and queer communities or are isolated may be underrepresented. Fifth, Vancouver had more follow‐up visits than Montreal and Toronto. Despite this difference, Engage had the same baseline and number of follow‐up months. However, a strength of RDS is the ability to recruit a more probabilistic community‐based sample and longitudinal data allowing us to estimate population‐level coverage over time.

## 5 CONCLUSIONS

Awareness and uptake of PrEP were much higher in Vancouver, likely indicating how free‐of‐charge public funding of PrEP in BC led to greater PrEP uptake compared with Ontario and Quebec, where PrEP is not fully free of charge. Future policies should consider providing full public funding for PrEP.

## COMPETING INTERESTS

None of the authors has any competing interests to declare.

## AUTHORS’ CONTRIBUTIONS

DMM, NJL, JC, GL, JJ, RSH and TAH designed the study. HA, AP and AL supervised data collection and study implementation. JB was responsible for managing the study database and developed the analytic dataset. SWN and SSS provided consultation on the analysis plan. LW conducted the analyses. JMS and KM developed the first draft of the manuscript and all authors provided input on updated versions. All authors have read and approved the final manuscript.

## FUNDING

Engage has been/is funded by the Canadian Institutes for Health Research (CIHR, #TE2‐138299, FDN‐143342, PJT‐153139), the CIHR Canadian HIV/AIDS Trials Network (#CTN300), the Canadian Association for HIV/AIDS Research (CANFAR, #Engage), the Ontario HIV Treatment Network (OHTN, #1051) and the Public Health Agency of Canada (#4500370314). TAH is supported by an Endgame Leader Chair Award in Gay and Bisexual Men's Health from the Ontario HIV Treatment Network. DMM and NJL are supported by Scholar Awards from the Michael Smith Foundation for Health Research (#5209, #16863). SSS is supported by a CIHR postdoctoral fellowship award. DG is supported by a Canada Research Chair in Sexual and Gender Minority Health. JMS is supported by a Michael Smith Foundation for Health Research Trainee award and a CTN CIHR Canadian HIV/AIDS Trials Network postdoctoral fellowship award.

## Data Availability

The data that support the findings of this study are available from the corresponding author upon reasonable request.
